# Computational Investigation of Voltage-Gated Sodium Channel β3 Subunit Dynamics

**DOI:** 10.3389/fmolb.2020.00040

**Published:** 2020-03-18

**Authors:** William G. Glass, Anna L. Duncan, Philip C. Biggin

**Affiliations:** Structural Bioinformatics and Computational Biochemistry, Department of Biochemistry, University of Oxford, Oxford, United Kingdom

**Keywords:** molecular dynamics, coarse-grain, epilepsy, lipid bilayer, multiscale

## Abstract

Voltage-gated sodium (Na_*v*_) channels form the basis for the initiation of the action potential in excitable cells by allowing sodium ions to pass through the cell membrane. The Na_*v*_ channel α subunit is known to function both with and without associated β subunits. There is increasing evidence that these β subunits have multiple roles that include not only influencing the voltage-dependent gating but also the ability to alter the spatial distribution of the pore-forming α subunit. Recent structural data has shown possible ways in which β1 subunits may interact with the α subunit. However, the position of the β1 subunit would not be compatible with a previous trimer structure of the β3 subunit. Furthermore, little is currently known about the dynamic behavior of the β subunits both as individual monomers and as higher order oligomers. Here, we use multiscale molecular dynamics simulations to assess the dynamics of the β3, and the closely related, β1 subunit. These findings reveal the spatio-temporal dynamics of β subunits and should provide a useful framework for interpreting future low-resolution experiments such as atomic force microscopy.

## Introduction

Voltage-gated sodium (Na_*v*_) channels are the initiators of action potentials in electrically excitable cells and are also implicated in many disease and pathological states including cardiac arrhythmia (Watanabe et al., 2008), epilepsy (Audenaert et al., 2003; van Gassen et al., 2009), neuropsychiatric disorders (Gargus, 2006), and chronic pain (Shah et al., 2000, 2001; Takahashi et al., 2003; Bouza and Isom, 2018). Na_*v*_ channels are comprised of an α subunit that forms the central pore-conducting region and β subunits that perform various roles such as modulating the voltage sensitivity and regulating the trafficking of the channel. In humans, there are ten α and four β subunits (the β1 subunit gives rise to two isoforms, β1 and β1B) that are expressed in different tissue-specific combinations, thus giving precise regio-selective control of the Na_*v*_ channel behavior. Additionally, β subunits also function independently as cell-adhesion molecules (CAMs) (Isom et al., 1995; Rougon and Hobert, 2003; Yu et al., 2003) and may play a role in Na_*v*_ channel clustering at the nodes of Ranvier (Ratcliffe et al., 2001) to promote the propagation of the action potential.

As perhaps might be expected given its central role in sodium ion conduction, most attention has been paid to the α subunit. However, *in vivo*, the effects of the β subunits are increasingly recognized and may well offer alternative therapeutic routes in the long run (Hull and Isom, 2018). For example, the β1 subunit has been shown to stabilize the Na_*v*_ 1.7 channel against mechanical stress (Körner et al., 2018) and has diverse roles with respect to its interactions with Na_*v*_ channels (Edokobi and Isom, 2018). People with mutations in the β3 subunit (*SCN3B gene*) show cardiac conduction problems (Brackenbury and Isom, 2011) and in mice deletion of SCN3B leads to cardiac arrhythmias (Hakim et al., 2008, 2010). The SCN3B gene has also been linked to Brugada syndrome (Okata et al., 2016).

Although the β1 – 4 isoforms all share a similar scaffold of an extracellular immunoglobulin (Ig) – like fold with a single transmembrane (TM) helix and unstructured intracellular domain, their binding to the α subunit differs. Both β2 and β4 bind covalently (Yu et al., 2003; Chen et al., 2012), via a disulfide bond, whilst β1 and β3 bind non-covalently. It has previously been shown that β3 subunits can trimerize via their Ig domains and can also induce higher order oligomerization of Na_*v*_ channel α subunits (Namadurai et al., 2014). Increasingly, there is evidence to suggest that sodium channels may in fact operate in higher order complexes (Clatot et al., 2017) and can also form complexes with many other proteins involved in a variety of signaling pathways (Kanellopoulos et al., 2018).

The α subunit itself is constructed from four homologous domains (DI – DIV), each containing six TM helices that make up the voltage sensor domain (VSD, helices 1 – 4) and pore-forming domain (helices 5 and 6). The exact location of where β subunits bind to the α subunit is uncertain, additionally the exact ratio of α:β subunit is also not very well characterized and may vary depending on tissue type and the cellular environment (Patino et al., 2011). Evidence from experimental fluorescence studies suggests that both the β3 and β1 subunits can bind to the α subunit and may alter the rate of fast inactivation through interaction with the VSDs of DIII and DIV, respectively (Zhu et al., 2017). Interestingly, recently released structures of Na_*v*_ channels with β subunits bound all contain β subunit density in this region. The first of the eukaryotic structures with a β subunit bound was that of Na_*v*_ 1.4 from Electric Eel by Yan et al. (2017), solved at a resolution of 4 Å. Here the fully resolved β1 subunit interacts via its transmembrane domain (TMD), and Ig domain with the VSD of DIII and extracellular loops of the α subunit, respectively. Shortly after, the nearly identical human structure of Na_*v*_ 1.4 was solved by Pan et al. (2018) with β1 again bound to the VSD of DIII at an improved resolution of 3.2 Å. In another cryo-EM structure, this time with the human Na_*v*_ 1.7 α subunit, not only could the position of β1 be resolved, but also the position of β2 and various toxin molecules (Shen et al., 2019). These structures offer an insight into not only the various states the α subunit occupies in its activation profile but also where β subunits may bind. In these structures the β1 subunit is bound to VSD of DIII, usually on the periphery of the α subunit. At this stage, it remains unclear as to whether the site of binding is consistent between α subunits or indeed whether the binding interactions for β1 will be the same for β3 (Zhu et al., 2017). Interestingly, it was recently reported that the human β1 subunit can also interact with the bacterial NaChBac channel (Molinarolo et al., 2018) although the mode of interaction was not discussed.

Despite the plethora of recent structural information, several aspects regarding the role of the β subunits remain unclear. What is the dynamic behavior of β subunit monomers? Do they oligomerize, and if so, how? How does the trimeric Ig domain structure of β3 relate to the position and orientation of the β subunits observed when in complex with the α subunits? To try and address these questions, we have used multiscale molecular dynamics simulations. We show that although β1 and β3 exhibit a relatively high sequence identity (51%), the behavior of the monomers is quite different, with β3 being more dynamic than β1. We attribute this to distinct residue – lipid contacts in the Ig domains of both subunits. We also demonstrate that the lipid composition is likely to have a key role in controlling the dynamical behavior.

## Materials and Methods

### Homology Models

#### β3 Monomer

The recent cryo-EM structure of the β1 subunit in the human Na_*v*_ 1.4-β1 complex (Pan et al., 2018) (PDB: 6AGF) was used to construct a model of the human β3 subunit. Sequence alignment was performed using the MUSCLE web server (Edgar, 2004) with the full length human β3 and the β1 cryo-EM structure sequence with a sequence identity = 51% (see Supplementary Figure S1 for sequence alignment and domain annotation). A total of 200 models were created with each model scored using Discrete Optimized Protein Energy (DOPE) in the Modeller software package (Webb and Sali, 2014). The 10 best models were ranked using Qualitative Model Energy Analysis (QMEAN) (Benkert et al., 2008) and the final model chosen with the highest QMEAN score.

#### β1 Monomer

The model used for β1 simulations was constructed directly from the Na_*v*_ 1.4-β1 structure (Pan et al., 2018), since all residues had been resolved. All mutations in the Ig domain and linker (see Table 1) were performed in PyMol (DeLano, 2004).

**TABLE 1:** Summary of simulations.

**Simulation**	**No. proteins**	**Bilayer composition**	**No. Lipids**	**Box size (x and y)**	**Duration**
**Atomistic**					
β1 monomer	1	POPC	225	9 nm	25 × 400 ns
β1 monomer (Ig mutant)	1	POPC	225	9 nm	3 × 400 ns
β1 monomer (Ig + linker mutant)	1	POPC	225	9 nm	3 × 400 ns
β1 monomer (linker mutant)	1	POPC	225	9 nm	3 × 400 ns
β3 monomer	1	POPC	225	9 nm	25 × 400 ns
β3 trimer	3	POPC	506	13 nm	3 × 400 ns
**Coarse-grained**					
β3	36	PM	10,080	52 nm	3 × 10 μs

#### β3 Trimer

The crystal structure of the trimeric β3 subunit (Namadurai et al., 2014) (PDB: 4L1D), containing just the extracellular region, was used as a template to construct a model of the trimeric extracellular region of β3. A total of 200 models were created with each model scored using DOPE in the Modeler software package (Webb and Sali, 2014). The 10 best models were ranked using QMEAN (Benkert et al., 2008) and the final model chosen with the highest QMEAN score.

#### Full Length β3 Trimer

Over the course of the simulations of the β3 monomer model (see section “β3 Monomer”) a large variety of conformations were visited. To construct the β3 trimer model a frame from the first run was taken at a pitch angle of 44.7° with the long axis of the Ig domain approximately perpendicular to the plane of the membrane. This was overlaid with each chain of the β3 crystal structure containing just the extracellular domain (ECD) (see section “β3 Trimer”). After the model was constructed it was checked for no steric clashes, of which there were none. All overlays were performed in PyMol (DeLano, 2004).

### Molecular Dynamics (MD) Simulations

All atomistic simulations were performed using GROMACS 2018 (Abraham et al., 2015) with the AMBER ff99SB-ILDN force field (Lindorff-Larsen et al., 2010). Protein models constructed with a membrane were prepared using the InflateGRO (Kandt et al., 2007) methodology and in-house scripts used for final adjustments. Equilibration steps of each system consisted of solvation using the TIP3P water model and neutralization using 150 mM NaCl, energy minimization using the steepest decent algorithm and a short (1 ns) and long (5 ns) equilibration whilst position restraining the Cα atoms with a force constant of 1000 kJ mol^−1^. All simulations were carried out in the NPT ensemble. The temperature and pressure were set to 300 K and 1 bar using the Nosé-Hoover thermostat (Nose, 1984; Hoover, 1985) and Parrinello-Rahman barostat (Parinello and Rahman, 1981) with coupling constants of 0.8 and 5.0 ps, respectively.

All coarse-grained (CG) simulations were performed using GROMACS (Abraham et al., 2015) 2019 with the MARTINI (v2) force field (de Jong et al., 2013). Each CG protein was embedded in a membrane using the INSANE (Wassenaar et al., 2015) methodology. For each system, energy minimization was performed with the steepest decent algorithm. Equilibration steps consisted of solvation using the non-polarizable MARTINI water model and neutralization using 150 mM NaCl, followed by a short (20 ns) and long (100 ns) equilibration whilst position restraining backbone atoms with a force constant of 1000 kJ mol^−1^. All simulations were carried out in the NPT ensemble at a temperature of 323 K and pressure of 1 bar. The V-rescale (Bussi et al., 2007) temperature and Berendsen pressure coupling (Berendsen et al., 1984) were used for short equilibrations with coupling constants of 1.0 and 8.0 ps, respectively. The V-rescale temperature coupling and Parrinello-Rahman pressure coupling were used for long equilibrations with coupling constants set to 4.0 ps and 8.0 ps, respectively. The 6 × 6 β3 grid was constructed by tiling a unit cell of one membrane embedded protein after the previously mentioned equilibration steps in the x and y direction.

All simulations performed are summarized in Table 1. All atomistic simulations were performed in a 1-palmitoyl-2-oleoyl-glycero-3-phosphocholine (POPC) bilayer whilst all CG simulations were performed in a generalized mammalian plasma membrane (PM) composition from Koldsø et al. (2014), where the composition of the membrane is as follows:

Upper leaflet: POPC(40):POPE(10):Sph(15):GM3(10): CHOL(25)
Lower leaflet: POPC(10):POPE(40):POPS(15):PIP_2_(10): CHOL(25)

Where POPE, 1-palmitoyl-2-oleoyl-glycero-3-phosphatidy lethanolamine; Sph, sphingomyelin; GM3, monosialodihexosylg anglioside; CHOL, cholesterol; POPS, 1-palmitoyl-2-oleoyl-glycero-3-phosphatidylserine; PIP_2_, 1-palmitoyl-2-oleoyl-gly cero-3-phosphatidylinositol-4,5-bisphosphate.

### Ig Orientation Analysis

Assessment of the Ig domain’s favored orientation was achieved by calculating the principal axes (PAs) at each frame and measuring the Tait-Bryan angles using the standard basis *e*_*x*_,*e*_*y*_,*e*_*z*_ as a reference [where *e*_*x*_=(1,0,0), *e*_*y*_=(0,1,0), and *e*_*z*_=(0,0,1)]. In order to calculate the PAs the center of mass was taken as the center of mass of secondary structures contained within the Ig domain (i.e., the β-sheets and 3–10 helices). This was chosen to minimize any noise associated with flexible loop movement over the course of the simulation. The PAs ***p**1, **p**2*, and ***p**3* were obtained via the diagonalization of the moment of inertia tensor, *I*.

(1) $$\text{I}=\sum_{i=1}^{N}m_{i}\,\left[\left({\text{r}}_{i}\cdot {\text{r}}_{i}\right)\,\sum_{\alpha =1}^{3}{\text{e}}_{\alpha }⊗{\text{e}}_{\alpha }-{\text{r}}_{i}⊗{\text{r}}_{i}\right]$$

(2) $$\text{ Λ }=U^{T}\,\text{IU}$$

Where *U* = (***p**1, **p**2, **p**3*) and Λ is a diagonal matrix of eigenvalues that correspond to the principal moments of inertia. At every frame, the first, second, and third principal axes were used to define a rotation matrix (based on the direction cosine matrix between each principal axis and the reference basis) and from this the Tait-Bryan angles computed. Using an intrinsic rotation formalism of ZYX the yaw, pitch, and roll angles were defined. In this study we focus on the pitch angle in relation to the Ig domain. All angle analysis was produced from in-house python scripts are available at https://github.com/bigginlab/protein_orientation.

## Results

### Dynamics of β1 and β3 Interactions With the Membrane

**FIGURE 1:**
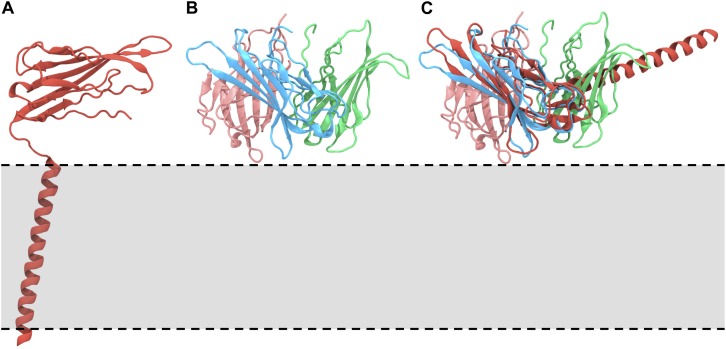
Orientations of the β1/3 subunit on the membrane. **(A)** The Na_*v*_ β1 subunit complex, highlighting the orientation, and interaction of the β1 subunit with respect to the membrane [PDB: 6AGF (Pan et al., 2018)]. **(B)** Structure of the trimeric Ig domain from β3 [PDB: 4L1D (Namadurai et al., 2014)]. **(C)** Overlay of the trimeric β3 Ig domain on the Ig domain of the β1 subunit, demonstrating the anticipated position of the β1 TMD and suggesting that these conformations are not compatible. The approximate location of the membrane is indicated by a gray box and dotted lines.

The recent structures of the Na_*v*_ α/β subunit complexes revealed the β1 Ig domain to adopt a conformation such that the long axis of the strands sits roughly parallel to the membrane surface (see Figure 1A). We noted at this point that if full-length β3 subunits adopted the same trimeric structure as observed for the Ig domain (from β3) only (Figure 1B; Namadurai et al., 2014), their interaction with the membrane would most likely require some substantial conformational rearrangement (Figure 1C). Thus, we investigated the dynamic behavior of full-length monomeric β1 and β3 in a POPC bilayer system using 25 replicas of 400 ns unbiased MD simulations.

**FIGURE 2:**
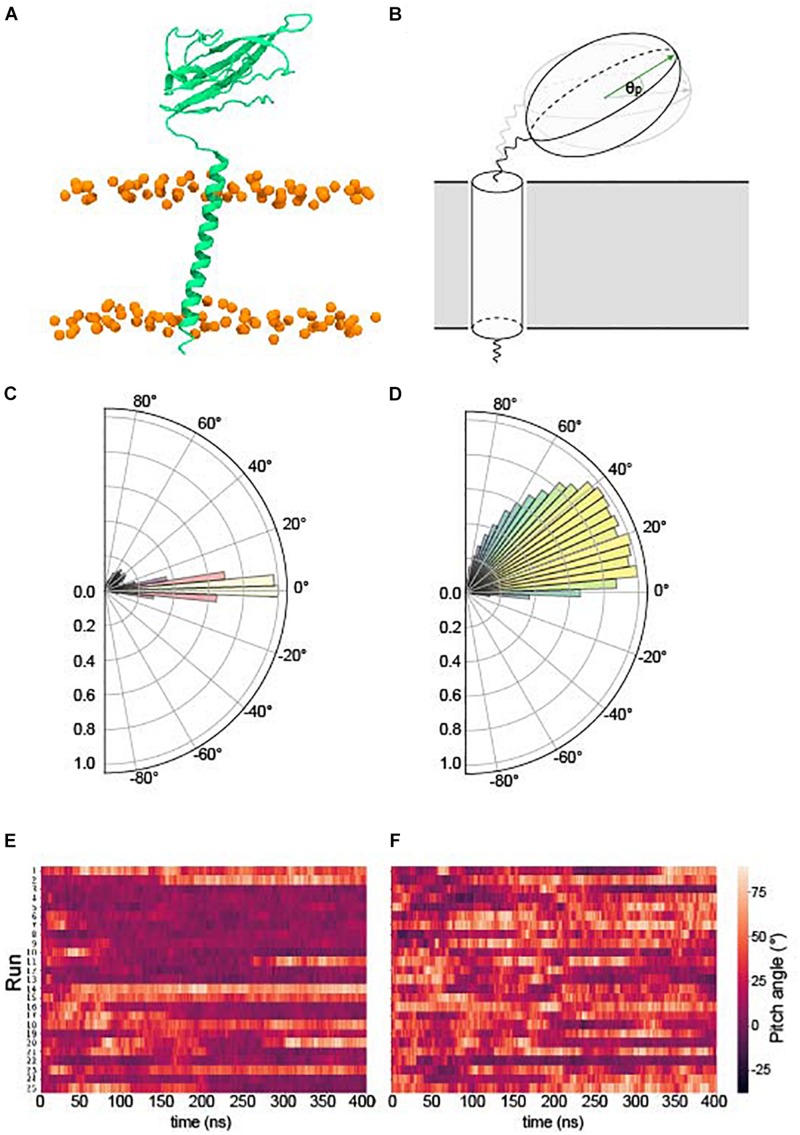
Pitch angles of the β1/3 Ig domain. **(A)** The starting conformation of both β1 and β3 subunits. **(B)** Schematic illustrating the pitch angle, θp (see section “Ig Orientation Analysis” for precise definition). **(C)** Histogram of the pitch angles visited of over 400 ns × 25 runs of the β1 – subunit. **(D)** Histogram of the pitch angles visited of over 400 ns × 25 runs of the β3 – subunit. **(E)** Heatplot of pitch angles over 400 ns in the β1 subunit. **(F)** Heatplot of pitch angles over 400 ns in the β3 subunit.

We examined the behavior of the Ig domain, in terms of the “pitch” with respect to the Ig domain in the first frame of each simulation (see section “Ig Orientation Analysis” and Figures 2A–F). Perhaps surprisingly, the behavior of the Ig domains in terms of the pitch is very different for β1 compared to β3 despite a high sequence identity (see section “β3 Monomer”). A pitch angle of 0°corresponds to an orientation parallel to the membrane plane and typically bound to the membrane surface. For β1 simulations, the pitch remains tightly clustered around 0°, with only a few runs exhibiting significant sojourns into higher pitch angles. In contrast, for β3 there is a wide variety of pitch states visited when analyzing all the repeats with a favored pitch angle centered around 30°. Individual runs (Figure 2F) also appear to show more dynamic movement of the Ig domain within runs.

**FIGURE 3:**
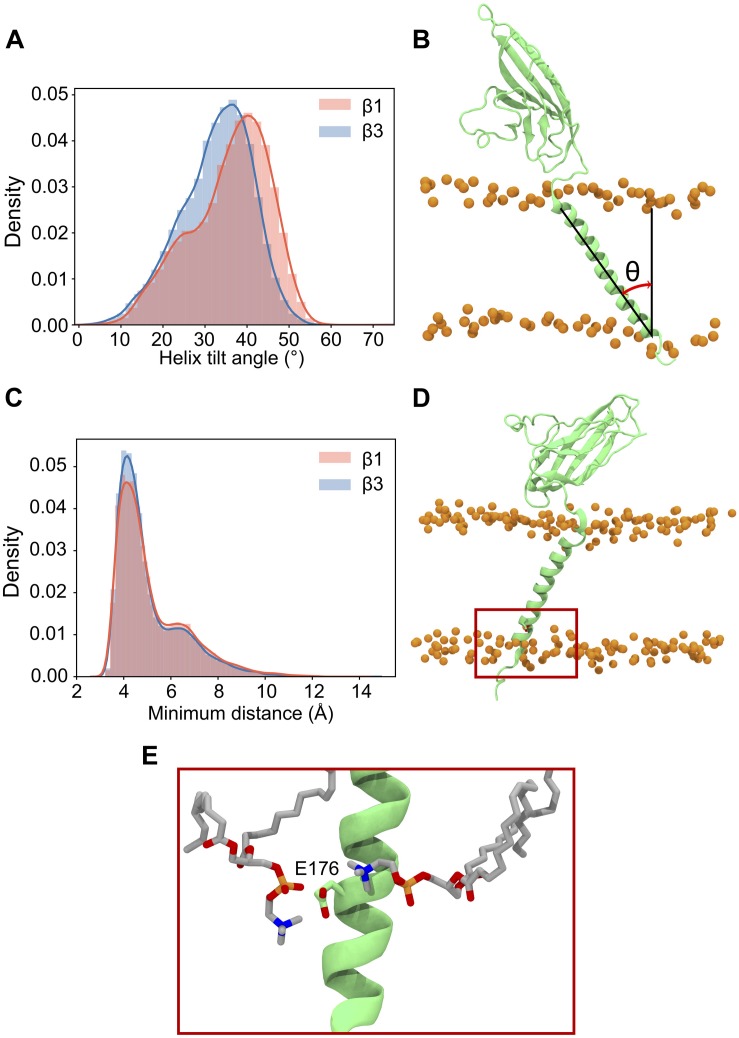
Tilting and position of E177(β1)/176(β3) in the β subunit transmembrane domain. **(A)** Histogram of TMD tilt angles over 25 × 400 ns simulations of the β1 (red) and β3 (blue) subunits. **(B)** Schematic of the angle used to measure the tilt angle in the TMD, phosphorus atoms of the POPC bilayer are shown as orange spheres. **(C)** Histogram of minimum distances between E177 (β1)/E176 (β3) (center of mass of sidechain oxygens) and the nitrogen atom of the surrounding POPC headgroups over 25 × 400 ns. The shoulder at a distance of 7 Å reflects the initial starting coordinates. **(D)** Position in the membrane of the conserved glutamic acid residue (highlighted inside a red box) in the β3 subunit after 400 ns. **(E)** Closer look at E176 (β3) in **(D)** with two nearby POPC residues interacting with the terminal oxygen atoms of the residue.

Our β3 model was constructed from the recent β1 cryo-EM structure (see section “β3 Monomer”) which, when bound to the α subunit, positions the TM helix approximately parallel to the bilayer normal. However, during simulations, both β1 and β3 TM helices adopt a significant tilt angle (Figures 3A,B), leading to a classic bell-shaped curve with a peak around 40° for β1 and 38° for β3. These are quite large tilt angles compared to many TM proteins (Bowie, 1997). Common to both β1 and β3 is a conserved glutamic acid residue (E177 and E176 in β1 and β3, respectively) that is located, somewhat surprisingly, within the lower part of the TM helix. Visual inspection of the trajectories suggested that it may play a role in maintaining the tilt angles. Analysis of the bilayer around this residue (Figures 3C–E) reveals that as the TM helix tilts there is a distortion of the membrane around E177/E176 in the lower leaflet where the carboxylic acid group of the side chain can interact with the positively charged NH_3_^+^ group of POPC.

**FIGURE 4:**
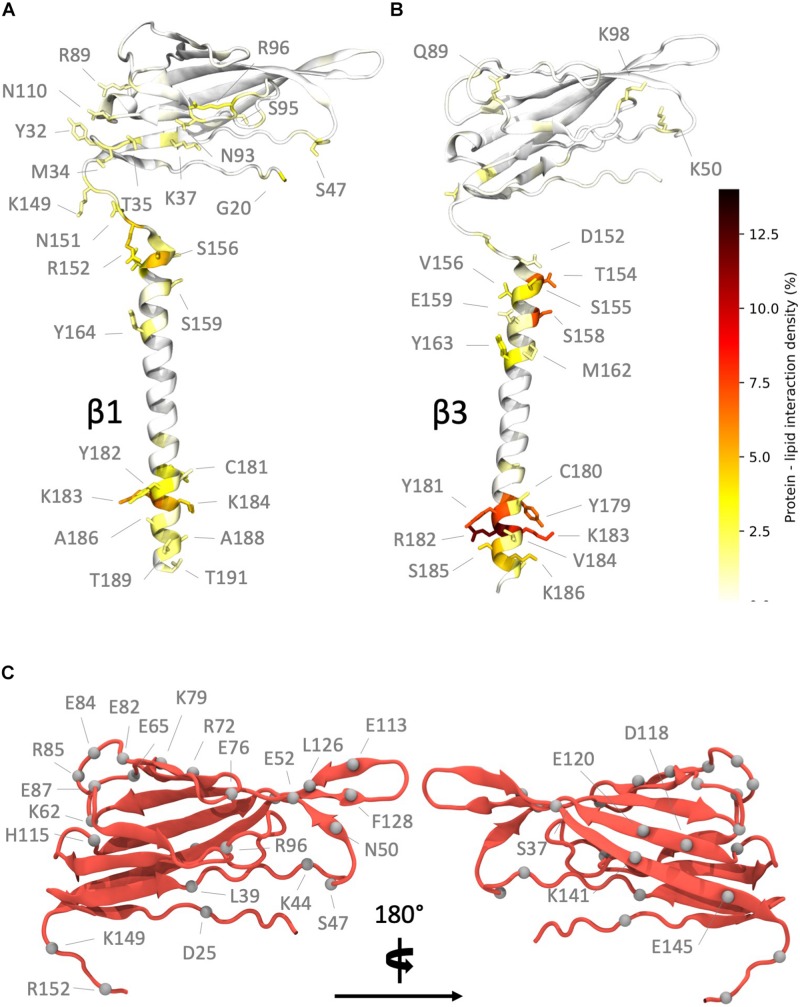
Regions of high interaction on the β subunit with a POPC membrane and non-conserved residues between subunits. **(A)** Regions of frequent interaction on the β1 subunit. **(B)** Regions of high interaction on the β3 subunit. The ECD of β1 exhibits more frequent regions of contact when compared to β3. **(C)** Non-conserved residues (shown as gray spheres) in the β1 subunit Ig and linker domains (some labels omitted in right hand image for clarity).

Further analysis of the contacts made between β subunits and the membrane (Figure 4) suggested that for β1 (Figure 4A and Supplementary Figure S2), the longest-lived interactions between the ECD and the membrane are, as perhaps might be expected, localized to polar residues and in particular, arginine and lysine residues. During analysis, a residue was considered to be in contact with the membrane surface if the center of mass of its side chain was within 5 Å of a phosphorus atom in the lipid headgroup. Protein – lipid contacts for each residue were calculated across all repeats and used to define the protein – lipid interaction density. The contacts seem to favor one “face” of the Ig domain, partially exposing the hydrophobic V27, V29, and P30 residues that are responsible stabilizing the observed Ig domain trimerization away from the Ig body in β3. For the β1 TM region, the longest-lived interactions are localized toward the end of the helix and again feature lysine residues K183 and K184 as well as Y164 and Y182.

In contrast, the Ig domain of β3 exhibits fewer regions of high contact, as expected from the analysis shown in Figure 2 where this domain exhibits orientations that place it away from the membrane surface. The contacts made (Figure 4B and Supplementary Figure S2), when in close proximity to the membrane, are again dominated by lysine and arginine residues (K50, K98, and R144). The longest-lived interactions from the TMD region are located at the intracellular end where a cluster of positively charged residues (R182, K183, and K186) interact with the phosphate headgroups. Interestingly these residues exhibit strong local bending, possibly due to the preference for these residues to interact with the bilayer.

At this point the extent of non-conserved residues was analyzed in both the β1 and β3 sequences, with a particular focus on charged residue differences in the Ig and linker domains between both subunits (Figure 4C). There are a total of 25 residue differences that are summarized in Table 2. In order to investigate the likely contribution that charged residues make to the observed differences between Ig domain orientation a series of systems were constructed (see Table 1 for simulation details). Firstly, the residues present in the β1 subunit Ig domain were mutated to the corresponding residue in β3 if they differed in charge, referred to as β1 Ig_*mut*_ hereafter. A second system was also prepared with two mutations in the linker (K149E and K152E), in addition to ones applied in the Ig domain (β1 Ig_*mut*_ + linker_*mut*_). Finally, a system with only the linker mutated (β1 linker_*mut*_) was prepared to assess what impact the linker has on β1 Ig domain dynamics.

**TABLE 2:** Charged residue differences between the β1 and β3 subunit Ig domains.

**Residue in β1**	**Equivalent residue in β3**	**Mutation performed**
**Ig domain**		
D25	P30	D25P
L39	R44	L39R
K44	M49	K44M
S47	E52	S47E
N50	E55	N50E
E52	T57	E52T
K62	E67	K62E
E65	K70	E65K
K69	I74	K69I
R72	–	R72A
E76	R78	E76R
E82	V84	E82V
E84	S86	E84S
R85	P87	R85P
E87	Q89	E87Q
R96	–	R96A
H115	D114	H115D
D118	L117	D118L
E120	T119	E120T
L126	E125	L126E
F128	E127	F128E
E133	R132	E133R
S137	K136	S137K
K141	L140	K141L
E145	R144	E145R
**Linker**		
K149	E148	K149E
R152	E151	K152E

**FIGURE 5:**
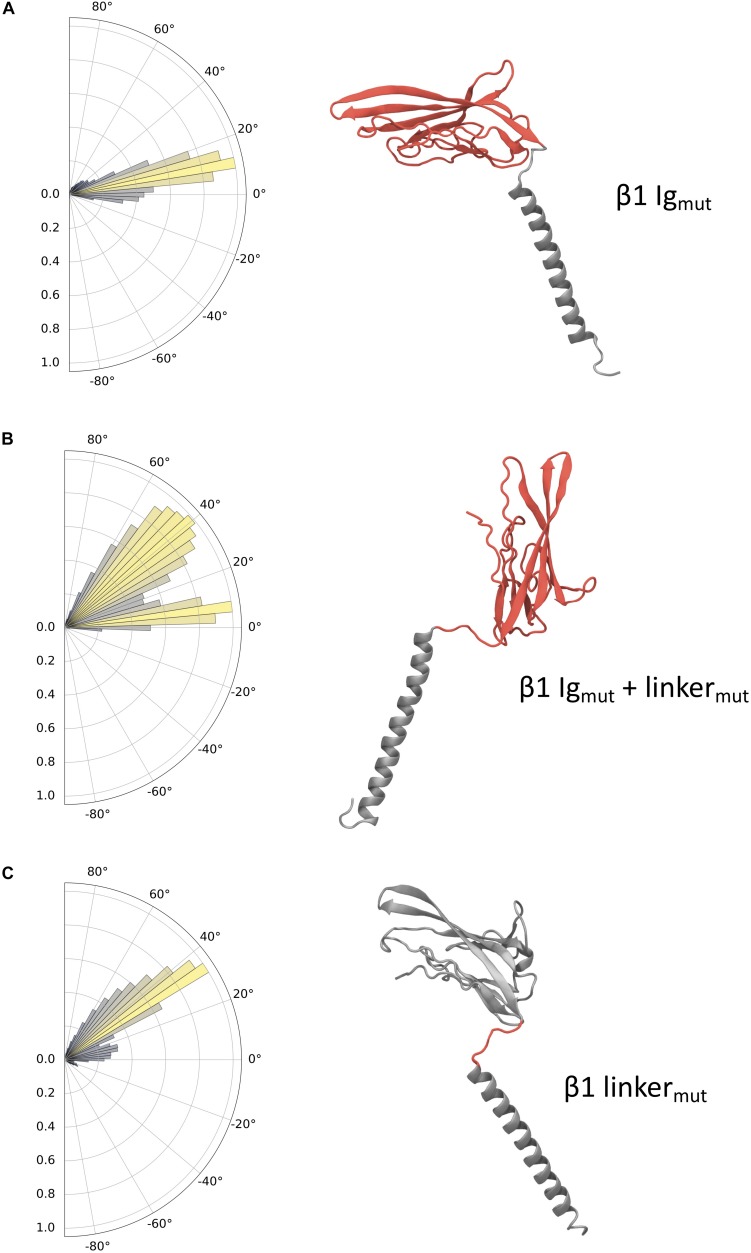
Ig domain dynamics in the mutated β1 subunit. Pitch angle analysis of the **(A)** β1 Ig_*mut*_, **(B)** β1 Ig_*mut*_ + linker_*mut*_, and **(C)** β1 linker_*mut*_. Snapshots of conformations for each system are shown with mutated domains indicated in red.

The effects of mutations in the domains of each β1 system (β1 Ig_*mut*_, β1 Ig_*mut*_ + linker_*mut*_, and β1 linker_*mut*_) reveal distinct dynamics (Figure 5) and hint at the regions responsible for differences observed in pitch angles between WT β1 and β3. The charge swaps within the Ig domain of β1 Ig_*mut*_ cause a slight increase in pitch angle to approximately 10° with respect to WT β1 (Figure 5A). In β1 Ig_*mut*_ + linker_*mut*_ the addition of K149E and K152E mutants in the linker drastically increase the sampled angles to values around 45°. Also present is another population close to WT β1 and β1 Ig_*mut*_ values, indicative of the Ig domain almost parallel to the membrane plane (Figure 5B). When applying only K149E and K152E in beta1 linker_*mut*_, the pitch angles populate values close to 40° with a smaller population at 10° reflecting an Ig domain pitch angle somewhere between membrane-bound and perpendicular to the membrane plane (Figure 5C). In addition to changes in Ig domain pitch with the K149E and K152E mutants there is also a tendency for the linker to become more linear as well as distinct changes in the Ramachandran plots at D148, located at the “hinge” before the start of the Ig domain (Supplementary Figure S3).

### Full-Length β3 Trimeric Model Dynamics

**FIGURE 6:**
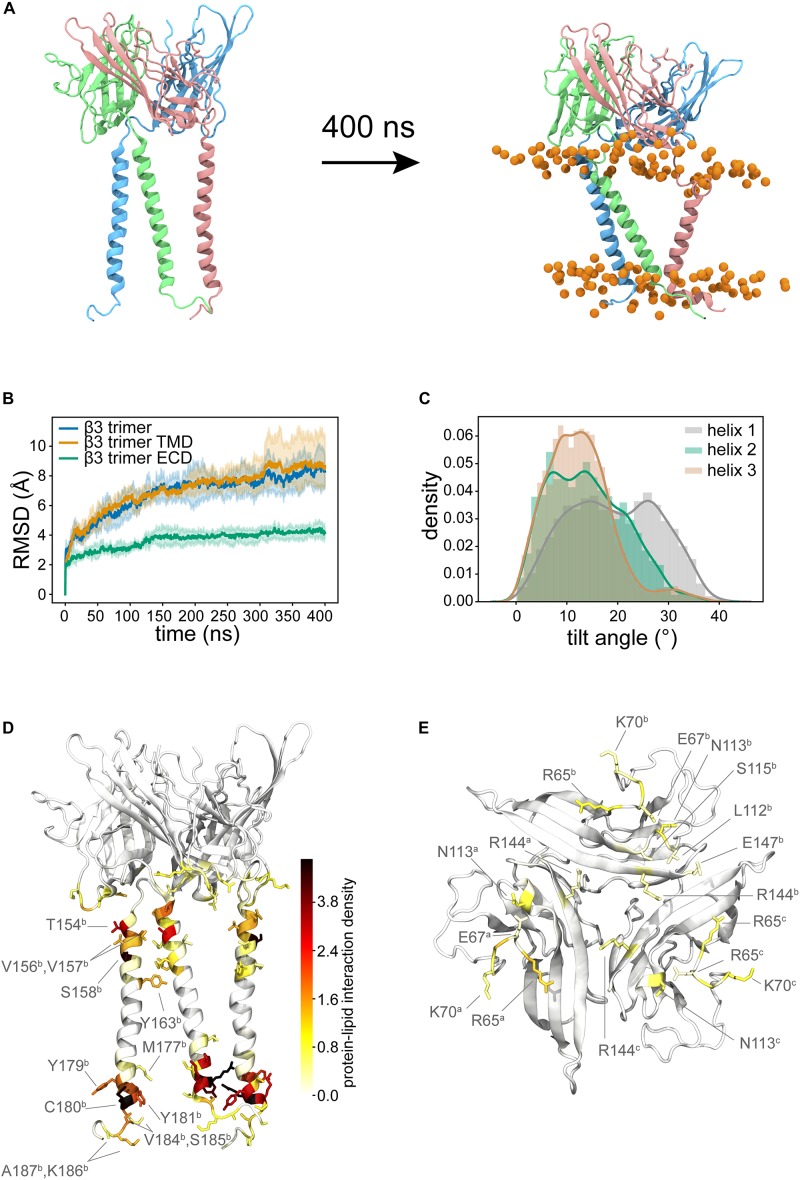
Trimeric model of full-length β3. **(A)** Shows the initial configuration with TM helices that almost parallel to the membrane normal. During the simulation, the TM helices adopt tilted orientations and the ECD domain continues to sit in a similar position to the initial configuration. **(B)** Average Cα RMSDs (from three runs) for the whole trimer (blue), the TM domains (orange, residues F153 to E189 of each subunit), the ECD only (green). Pale background reflects one standard deviation. **(C)** Distribution of tilt angles for the three helices in the trimer. **(D)** Probability density colored from white to red to black mapped onto the structure to show the lipid-protein interactions. **(E)** shows the key residues of the ECD that form interactions with the membrane. In both **(D,E)** different protein monomers are indicated in superscript.

Recently there have been several high-quality cryo-EM structures of full length β subunits bound to the α subunit of Na_*v*_ channels (Yan et al., 2017; Zhu et al., 2017; Pan et al., 2018; Shen et al., 2019). However, the trimeric crystal structure of β3 (Namadurai et al., 2014) lacks the TMD and its role, if any, to observed β subunit clustering remains elusive. To investigate the role of the TMD in β3 – β3 interactions and vice-versa, a trimeric model was constructed using the ECD β3 trimer and the TMD of the β3 monomer (see section “Full length β3 Trimer”). A total of three repeats of 400 ns atomistic MD were performed. As expected the extracellular trimeric structure remained intact and conformationally stable throughout the simulations (Figures 6A,B) and remained in an “upright” position on top of the membrane surface. The TM helices on the other hand were much more mobile (and indeed dominate the overall Cα root mean squared deviation (RMSD) (Figure 6B). Visual inspection of the trajectories revealed that the TM helices exhibit considerable lateral movement with respect to each other and appear to adopt significant tilt compared to the starting conformations. Analysis of the helical tilt angles (Figure 6C) confirms the adoption of significant tilt but also reveals that the helices can adopt a range of different tilt angles with a significant proportion centered around ∼ 12° and another around ∼ 26°. Even though there are strong preferences for these particular tilt angles, each helix is still able to visit the whole range of tilt angles from 0 to just over 40°. Note that for monomeric β3 (Figure 3A) the distribution was a classic bell-shaped curved centered around 38° suggesting that in the trimer, the tilt angle is, as might be expected, restricted by the tethering to the ECD.

We next analyzed the interaction of the protein with the lipid membrane. The interactions in the TM region (Figure 6D and Supplementary Figure S4) are very similar to those observed for the β3 monomers. There was also a significant amount of interaction between the bottom face of the ECD and the membrane (Figure 6E and Supplementary Figure S4), mediated in the main by positively charged residues, but not exclusively so by any means.

### Clustering of β3 Subunits in a Realistic Membrane Model

**FIGURE 7:**
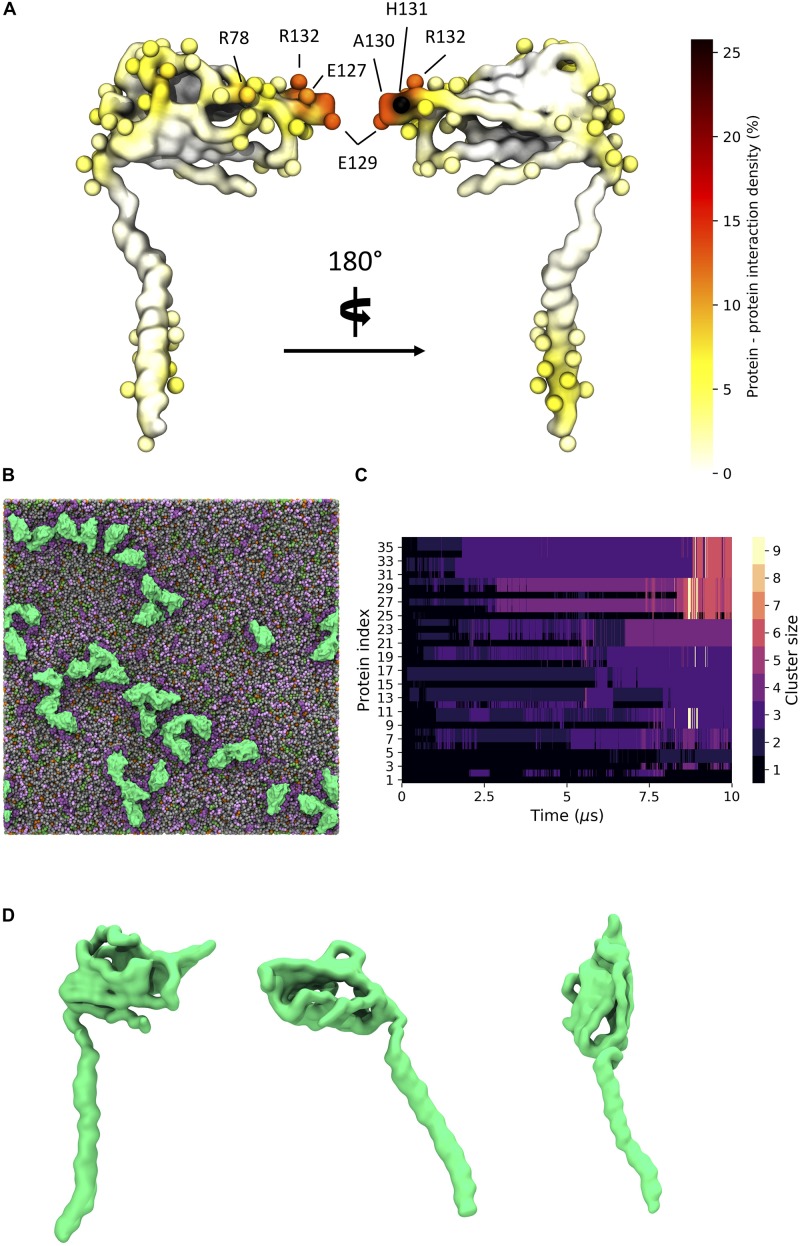
β3 clustering in a general mammalian membrane. **(A)** Regions of high protein - protein contact visualized on the β3 subunit surface colored as a probability undergoing an interaction with another protein. Spheres indicate residues with total interactions above 2.5% of the total time. **(B)** Typical clustering of β3 subunits (green) in a mixed lipid membrane (viewed from the extracellular side). Lipids visible in the upper leaflet include POPC (gray), POPE (green), Sph (pink), GM3 (purple), and Chol (orange). **(C)** Evolution of β3 clusters over a 10 μs simulation. Lighter colors indicate higher order clusters. **(D)** Distinct conformations of the β3 subunit involved in clusters. From left to right: down, intermediate, and up states.

Given the recent observation from atomic force microscopy (AFM) that β3 monomers could aggregate and form higher-order oligomers including dimers and trimers (Namadurai et al., 2014), we set up CG MD simulations to investigate how such oligomers might come together [see section “Molecular Dynamics (MD) Simulations”]. We set up a large membrane with a composition that replicated an endothelial cell (Figure 7A) and inserted 36 copies of the β3 subunit model and ran three independent simulations for 10 μs each. β3 subunits were indeed observed to form high-order oligomers (Figure 7B). The size of the clusters was analyzed over the course of each run and it was found that the cluster size tended to be present as a monomer or dimer with a significant population of higher order clusters (Figure 7C).

Long, fibril-like structures were formed in all repeats, with the Ig domains often making tip-tip interactions in a manner reminiscent of the interactions between the DIP and Dpr neuronal recognition proteins (Cosmanescu et al., 2018). Protein – protein contacts were measured over the three repeats. There are typically high regions of interaction on the last ∼10 residues in the TMD region of the β3 subunit as well as contacts present in the Ig domain. High regions of contact include residues 128 – 135 that correspond to the FEAHRPFV loop, at the “tip” of the Ig domain, located between the F and G β strands (see Supplementary Figure S1 for strand labeling) of the Ig domain (Figure 7A and Supplementary Figure S5). There are also regions of interaction in the loop region of residues 79 – 82 (NGHQ) and 89 – 92 (QGRL) between β strands C” and D that form one face of the Ig domain (Figure 7A). At the C-terminus of the TMD, residues M177, C180, Y181, K183, and V184 show regions of increased interaction between subunits. Further investigation of the Ig domains orientation on the membrane surface revealed a variety of conformations that reflect the dynamics seen in atomistic simulations. A number of protein copies were present with the long axis of the Ig domain parallel to the membrane whilst another population showed the Ig domain pointing up and away from the membrane, similar to the orientation seen in the trimeric crystal structure (Figure 7D).

**FIGURE 8:**
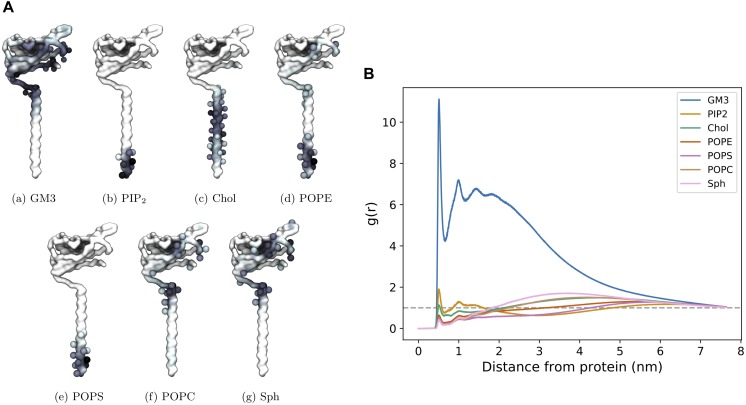
CG β3 interactions in a mixed lipid membrane. **(A)** Protein – lipid interactions visualized on face 1 of the β3 model. The first side chain particle of residues that account for over 2.5% of the total interaction time are shown as spheres. **(B)** Radial distribution function of the distribution of lipids surrounding β3 subunits in the PM.

We also investigated protein – lipid contact sites. Interactions were counted using the headgroup bead of each lipid type and a cut-off value of 6.5 Å. It can be seen (Figures 8A,B) that there is a slight preference for one side (which we label Face 1) of the Ig domain to interact with the lipid membrane, most notably for GM3. The other side (Face 2) of the Ig domain retains interactions with GM3 but to a lesser extent than Face 1 (Supplementary Figure S6). The radial distribution function reflects the high levels of interaction with GM3 as well as with PIP_2_ and cholesterol where the latter two interact with the TMD.

## Discussion

### β Subunit Monomers Exhibit Distinct Differences

Although similar in sequence and underlying fold, the behaviors of the β1 and β3 subunits in the membrane exhibit some striking differences. Our simulations suggest that the ECD of the β3 subunit is much more dynamic than β1. In contrast to the β1 subunit structure from Pan et al. (Pan et al., 2018), the Ig domain of the β3 subunit samples several pitch states (see Figure 2), with only a few corresponding to the β1-like cryo-EM structure. Conversely, the β1 subunit simulations provide evidence for a more restricted Ig motion, with the long axis of the Ig domain parallel to the membrane plane in 60% of the simulations performed. This increased membrane interaction may go some way to explain why β1 has a decreased propensity to form higher order oligomers, since the Ig domain is restricted to lie close to the membrane surface. The interaction in the β1 cryo-EM structure (Pan et al., 2018) between the ECD and the top of the VSD of DIII involves the conserved C21 – C43 disulfide bond. This orientation of the ECD in this cryo-EM structure is quite similar to the orientation we observe for β1 monomers in the membrane and thus we hypothesize that a monomer moving from the membrane to interact with an α subunit would only require a small change in conformation. Clearly, electrostatic interaction between the Ig domain and membrane surface will contribute to the preferred Ig orientation that both β1 and β3 adopt. Charge swap mutations in the β1 subunit for those present in β3 supports this (see Table 2 and Figure 5). Mutations performed within the Ig domain have a small effect on Ig domain pitch, however, the addition of two mutations (K149E and K152E) in the linker cause β3 Ig domain dynamics to be partially recovered in β1. The linker’s contribution to Ig dynamics is somewhat reduced when only K149E and K152E mutations are present in β1 and suggests that, although important, the difference in dynamics between both β1 and β3 may be a compound effect within both the Ig and linker domains of both subunits.

### The Transmembrane Helix Undergoes a Large Tilt

In both the β1 and β3 models there is a slight shortening of the TMD as well as the large helical tilt in the membrane. In the cryo-EM structure (Pan et al., 2018), this region of the β1 subunit has the lowest resolution of around 4.2 Å and we hypothesize that the TMD region of the β subunit may indeed be flexible until any hydrophobic mismatch with the bilayer is optimized either by TM helix tilting or bending. The tilting in both β1 and β3 is facilitated in part by the presence of a glutamic acid (see Figure 3). This glutamate is highly conserved and is found in both β1 and β3 sequences. Given its unusual position, it has been argued that it is likely to have functional significance and indeed has been investigated within β1 (McCormick et al., 1999) and β3 (Namadurai et al., 2014; Salvage et al., 2019). It also seems that for a full-length model based upon the trimeric β3 crystal structure of the ECD to be adopted in the context of a lipid bilayer system, the TMD helices of our model must change their tilt with respect to the membrane normal.

### Behavior of the Trimer

A major difference between monomeric and trimeric β3 lipid interactions is in the Ig domain. As part of a trimer, the Ig domain is no longer able to sample large pitch states due to the favorable hydrophobic interactions in the N-terminus of each chain. As such, the lipid interaction between the Ig trimer is markedly reduced when compared to the monomer with only a few charged residues (R65, E67, and K70) interacting with the membrane surface. Interestingly if the β3 subunit were to interact with the VSDs of the pore-forming α subunit in a similar fashion to β1 there would need to be substantial rearrangement of the Ig domains. This leads to the question of what, if any, the role of the TMD helices could play in α - β and/or β - β interactions? Lipid contact analysis in the TMD reveals that there is little difference between the trimeric and monomeric models. Close to the Ig domain, the restriction imposed via the stable Ig trimer reduces translational motion, whereas at the intracellular end the translational motion is much more dynamic with no clear preference for residue – residue interaction between chains. These results suggest that the TMD of the β3 subunit does not have an overall stabilizing effect on the β3 trimer and in fact may only be required for correct positioning within the membrane. This is in agreement with previous super-resolution microscopy data, where the density function estimated from the C-terminal mEos2-tagged β3 was consistent with a relatively unconstrained transmembrane helix/C terminus. This suggests that any trimerization events are likely to be controlled via the Ig domain. Additionally, when the helices of each β3 chain are in close proximity, the conserved E176 residue appears to be orientated away from the trimer center and preferentially interacts with the POPC membrane (data not shown). These observations are also supported by recent experimental work examining the role of E176 in β3 subunits, and that also concluded that oligomerization was dependent on the extracellular domain but not E176 (Salvage et al., 2019).

### β3 Subunit Clustering

It has been previously reported (Namadurai et al., 2014) via the use of AFM and Fluorescence Photoactivated Localization Microscopy (FPALM) that the β3 subunits can form higher order oligomers. In particular, the AFM suggested the presence of dimers and trimers, whilst the FPALM experiments suggested the presence of a trimer in live cells. In our large CG simulations, where we try to capture the complexity of a mammalian cell membrane, we do indeed observe the formation of oligomers. The interactions between individual β3 subunits tends to show an “end on” interaction, whereby the tip of one Ig domain interacts with the base of another to produce long, fibril-like oligomers with a small contribution from the C-terminal end of the TMD. This leads to a slightly different picture of how the β3 subunits may interact compared to that arrived at by Namadurai et al. (2014) who interpreted the formation of the trimers in the context of a crystal structure of the β3 Ig domain (and forms distinct trimers). The formation of similar, but full-length, trimers would mean that the Ig domains must frequently “lift off” the surface of the membrane (see Figure 5A) and the oligomerize predominantly through the exposed flat face of the Ig domain. Although we observe movements of the Ig domain in the atomistic simulations (see Figure 2D) that would be compatible with the formation of such a trimer, we observe such movements in the CG simulations only infrequently. Furthermore, such orientations (Figure 6) are too short-lived relative to the time required for oligomerization via the exposed faces of the β-sheets. A key difference to note here is that the atomistic simulations were performed in a POPC bilayer, whereas the CG simulations were performed in a bilayer of a more complex composition. Ideally the use of a mixed lipid membrane at an atomistic level would reveal finer protein – lipid interaction details. However, to study large scale clustering would require computational resource beyond our current capability. Visual inspection of the CG simulations suggests that the interaction of the Ig domain with the headgroup of the GM3 headgroup appears to keep the Ig domain close to the surface of the membrane. On the face of it, this may appear at odds with the interpretation by Namadurai et al. (2014). However, there is no direct atomically detailed evidence of how the full-length β3 subunit may come together, and the work here, presents an alternative possibility. Regardless, the results here suggest that spontaneous oligomerization of a full-length trimer, where the Ig domains adopt the crystal structure conformation, would likely be a very slow process if it does occur.

## Conclusion

In this work, we have explored the dynamics of the β1 and β3 subunit monomers with a lipid bilayer. The dynamics exhibited a remarkable and unexpected difference in behavior of the ECD, which we attribute to distinct binding patterns within the Ig domain. It will be interesting to investigate the influence of the non-conserved charged residues between both subunits in future experiments. A full-length model of a β3 subunit based on a trimeric structure of the Ig domain only, suggests that the TM helices do not interact particularly strongly. Finally, the CG simulations suggest that higher order oligomerization of monomers may be mediated by “end-on-end” interactions. These results should provide a useful framework on which to interpret low-resolution methods such as AFM that are examining the nature of oligomerization in ion channels. The existing agreement between experiment and simulation is encouraging.

## Data Availability Statement

All datasets generated for this study can be obtained via request to the corresponding author.

## Author Contributions

WG performed and analyzed all simulations and co-wrote the manuscript. AD performed analysis and gave advice. PB conceived the work and co-wrote the manuscript.

## Conflict of Interest

The authors declare that the research was conducted in the absence of any commercial or financial relationships that could be construed as a potential conflict of interest.
