# Supplementary material for: Computational Investigation of Voltage-Gated Sodium Channel β3 Subunit Dynamics
Source: Front Mol Biosci. 2020 Mar 18;7:40. doi: 10.3389/fmolb.2020.00040 (PMC7103644; doi:10.3389/fmolb.2020.00040)
Supplement: Supplementary file 1 [file Presentation_1.pdf]

Supporting Information

Computational Investigation of Voltage-gated Sodium Channel β3 Subunit Dynamics

William G. Glass, Anna L. Duncan, and Philip C. Biggin*

Structural Bioinformatics and Computational Biochemistry, Department of Biochemistry, University of Oxford, South Parks Road, Oxford, OX1 3QU, United Kingdom.

*To whom correspondence should be addressed.

Email: philip.biggin@bioch.ox.ac.uk

Tel. +44 1865 613305

Fax. +44 1865 613238

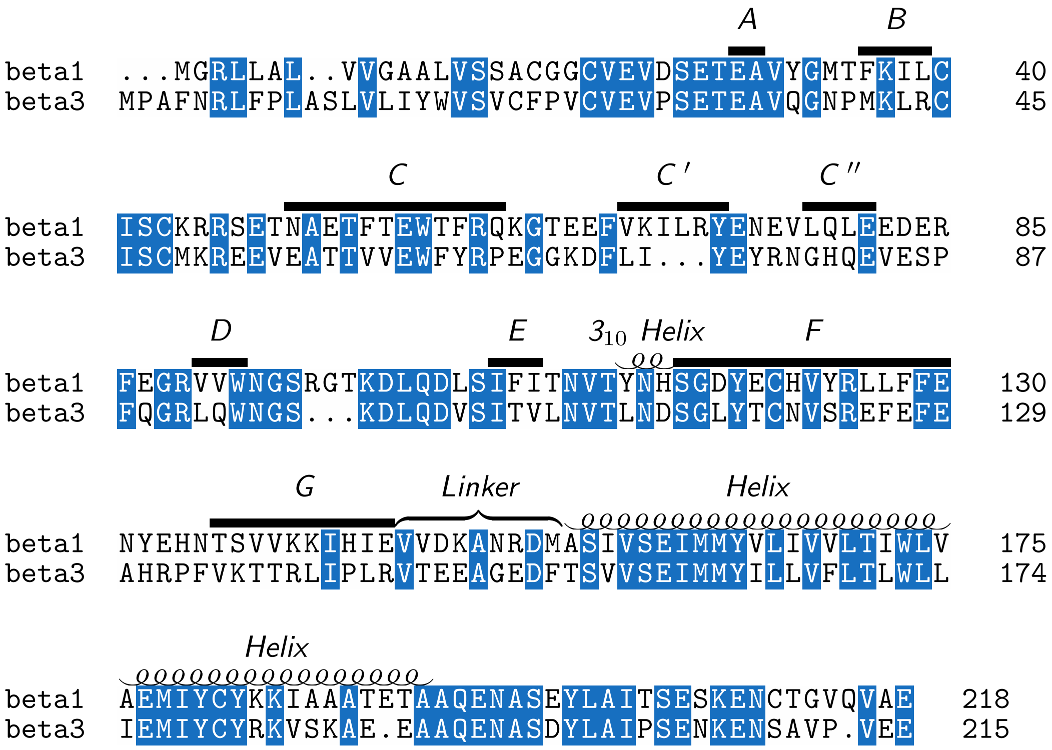

Figure S1. Sequence alignment between human β1 & β3. The approximate locations of domains are indicated: β strands in the Ig domains are shown as black rectangles (labelled A – G, with C' and C'' indicating the additional hairpin present in V-type Ig domains), helical regions are represented by spirals and the short linker region between the Ig domain and transmembrane domain is indicated with curly braces. Shaded blue regions correspond to 100% sequence identity.

A

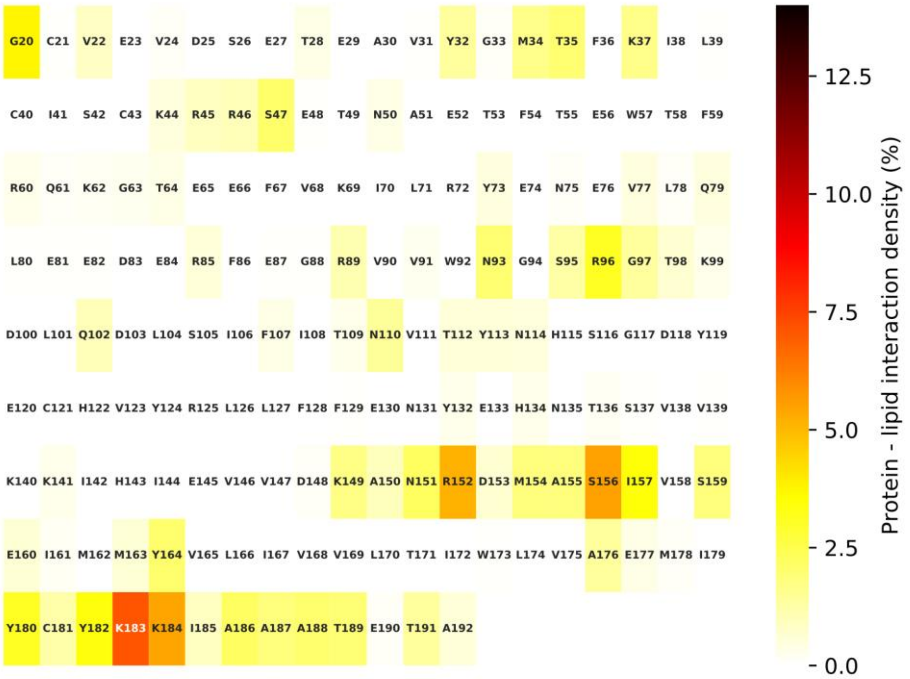

B

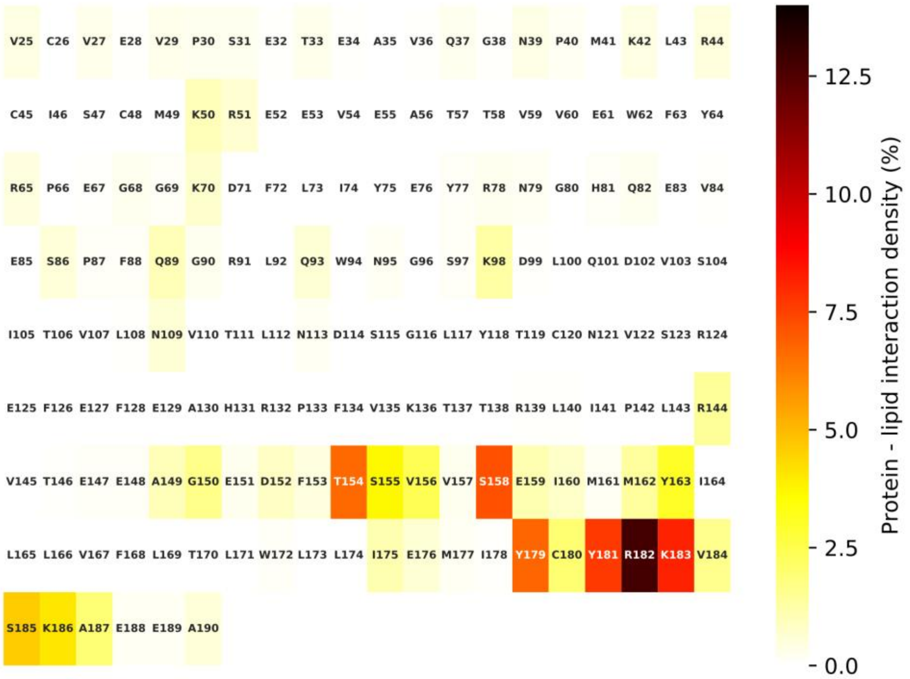

Figure S2. β1 & β3 atomistic monomer average contact maps with the phosphate headgroup of a POPC membrane. (A) β1. (B) β3.

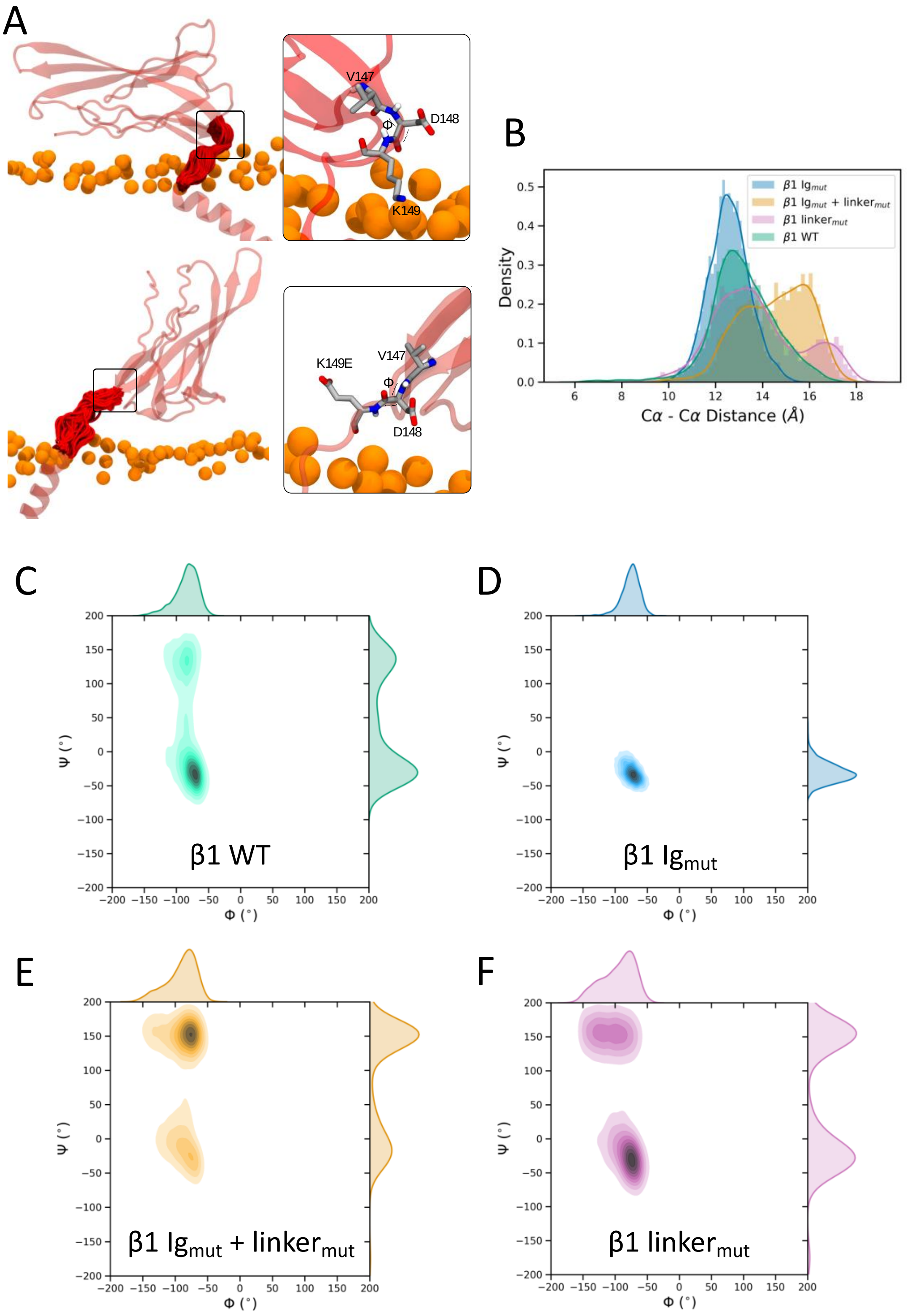

Figure S3. **(A)** Typical conformation of both WT β1 and the β1 Ig$_{mut}$, exhibiting linker bending around D148 (top) and a typical conformation of the β1 Ig$_{mut}$ + linker$_{mut}$ with a more linear and disordered linker (bottom). Snapshots of the linker are shown in deep red with the residues surrounding D148 shown in the inset with ϕ and φ angles indicated. **(B)** Histograms of the Cα – Cα distance in the linker (defined between V146 and R/E152), where mutation of the linker causes an average increase in linker length. **(C - F)** Ramachandran plots of the D148 backbone in β1 WT, β1 Ig$_{mut}$, β1 Ig$_{mut}$ + linker$_{mut}$, and β1 linker$_{mut}$. Upon mutation of the Ig domain and linker there is an increase in sampling of φ angles around 150°, this trend is followed to a lesser extent with only the linker mutated (i.e. K149E and K152E).

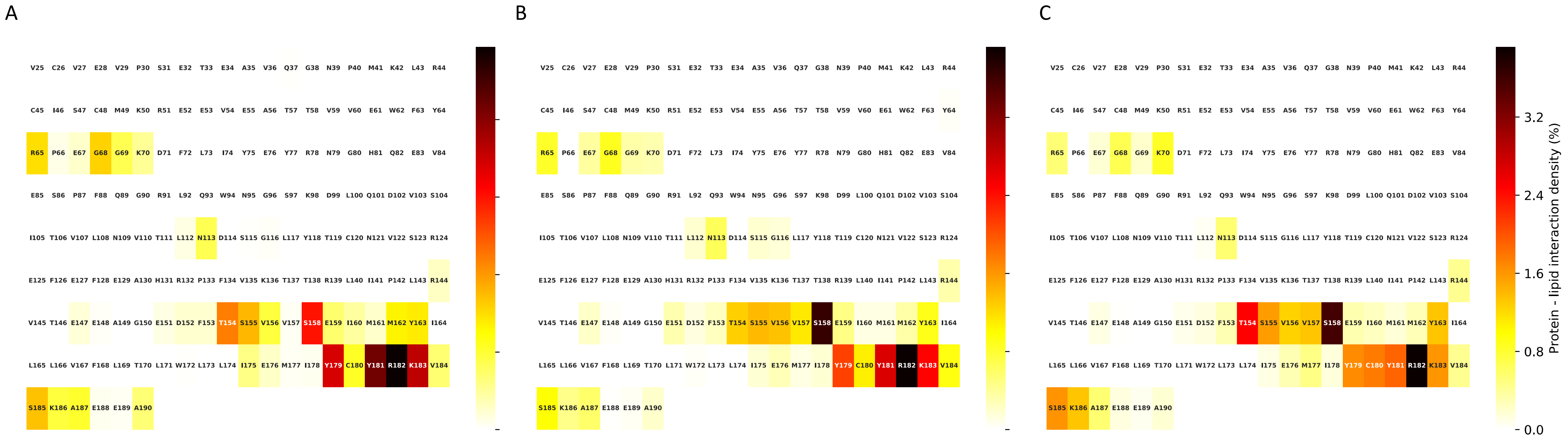

Figure S4. β3 trimer – POPC contact maps. (A) – (C) represent averages across each β3 chain in the trimeric construct.

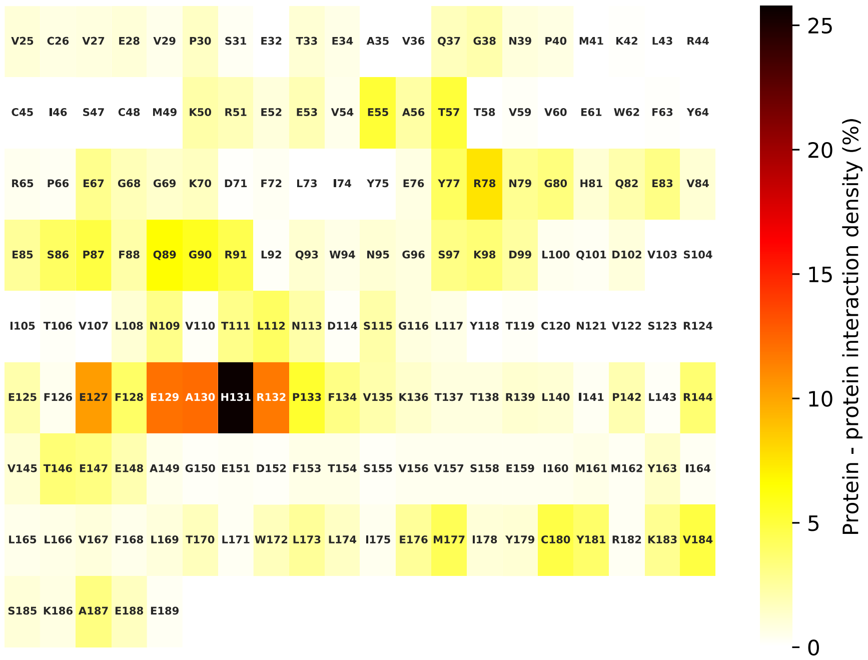

Figure S5. Heat map for the protein – protein contacts between copies of the coarse-grained β3 system.

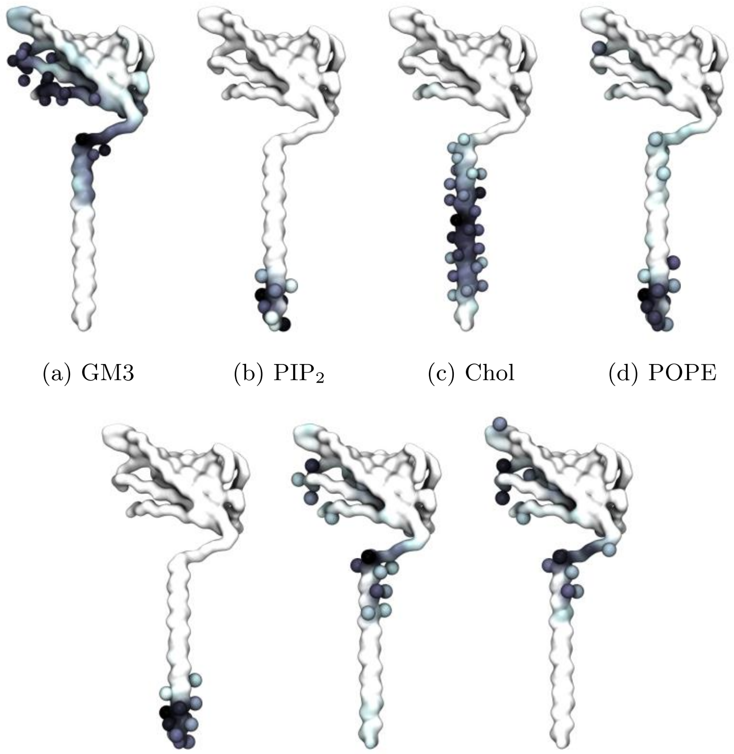

(a) GM3 (b) PIP_2 (c) Chol (d) POPE

(e) POPS (f) POPC (g) Sph

Figure S6. Protein-lipid interactions on Face 2 of the coarse-grained β3 subunit.
